# Physical, Cognitive, Social, and Functional Health Correlates of Major Depressive Disorder Subtypes: A Systematic Review

**DOI:** 10.3390/brainsci15050525

**Published:** 2025-05-19

**Authors:** Jen E. McKeough, Christopher F. Sharpley, Kirstan A. Vessey, Vicki Bitsika, Rebecca J. Williams, G. Lorenzo Odierna, Ian D. Evans

**Affiliations:** Brain-Behaviour Research Group, University of New England, Armidale, NSW 2351, Australia; jborrell@une.edu.au (J.E.M.); kvessey@une.edu.au (K.A.V.); vicki.bitsika@une.edu.au (V.B.); rwilli90@une.edu.au (R.J.W.); godierna@une.edu.au (G.L.O.); ievans3@une.edu.au (I.D.E.)

**Keywords:** depression, major depressive disorder, subtypes, health effects

## Abstract

Background/Objectives: Major Depressive Disorder (MDD) is a neuropsychiatric disorder affecting nearly 200 million people worldwide. While it has broad health effects, relatively little is known about how these vary across MDD ‘subtypes’, which reflect distinct symptom profiles. This systematic review examined the methods used to define several MDD subtypes and their associations with physical, cognitive, social, and functional health outcomes. Methods: A systematic search of PubMed was conducted in accordance with PRISMA 2020 guidelines to identify peer-reviewed studies published in English between 2014 and 2025. The final search was conducted on 21 January 2025. Studies were included if they examined adults with MDD subtypes and reported health-related outcomes. Risk of bias was assessed using the Newcastle–Ottawa Scale. A narrative synthesis was conducted due to heterogeneity in the subtype definitions and outcome measures. Results: Sixteen studies were included. Atypical and melancholic depression were most consistently associated with metabolic dysfunction, higher BMI, and a greater waist circumference. Melancholic depression was frequently associated with cognitive deficits, though results varied. Cognitive impairments were also observed in DSM-defined atypical depression, particularly in attention, vigilance, and social cognition. Anxious and melancholic depression may be associated with more severe social and functional impairment compared to other subtypes. However, the findings were limited by inconsistent definitions and outcome measures. Conclusions: Some subtypes, particularly atypical, melancholic, and anxious depression, are differentially associated with specific patterns of impairment, though inconsistencies limit firm conclusions. Registration: This review was retrospectively registered with the Open Science Framework (OSF): No specific funding was received.

## 1. Introduction

### 1.1. Major Depressive Disorder (MDD)

MDD is a pervasive mental health condition affecting over 280 million individuals worldwide [1], making it a leading cause of disability and a major contributor to the global burden of disease [2]. Beyond psychological symptoms, MDD has wide-ranging effects on physical health [3,4,5], cognitive abilities [6], social relationships [7], functional capacity [8], and quality of life (QoL) [9,10]. These broad impacts underscore the complexity of MDD and highlight the importance of refining classification approaches that capture individual differences in symptom presentation and associated health outcomes.

Despite being diagnosed as a single disorder, MDD is highly heterogeneous, with substantial variation in symptom expression, illness trajectory, and treatment response [11,12,13,14]. Current diagnostic frameworks, such as the Diagnostic and Statistical Manual of Mental Disorders, Fifth Edition, Text Revision (DSM-5-TR) [15] and the International Statistical Classification of Diseases 11th Revision (ICD-11) [16] define MDD based on a minimum number of required symptoms. However, when the nine Diagnostic Criteria and the Associated Features of MDD are considered, a possible 1497 symptom combinations meet the diagnosis for MDD [17,18]. This variability in symptom profiles means that two individuals with MDD may present with largely different symptom profiles, raising concerns about the clinical utility of a ‘one-size-fits-all’ approach to treatment.

To address this symptom diversity, various subtyping approaches to MDD have been proposed. A commonly used approach defines MDD subtypes using DSM specifiers, which are optional descriptors applied to a primary MDD diagnosis such as ‘with melancholic features’, ‘with atypical features’, ‘with mixed features’, and ‘with anxious distress’ [15]. These specifiers indicate specific symptom patterns, for example, depression with melancholic features is characterised by anhedonia, psychomotor disturbances, and neurovegetative symptoms [19], while depression with atypical features is associated with mood reactivity, hypersomnia, and increased appetite [20]. Specifiers are often used in research to distinguish between MDD subtypes [15,21,22,23]. Alternatively, data-driven methods such as latent class analysis (LCA) and cluster analysis have been used to empirically derive subtypes based on underlying symptom patterns [24,25].

Despite the increasing recognition of MDD subtypes in research and clinical practice, it remains unclear whether these classifications reliably differentiate patients in clinically meaningful ways in terms of their broader health. For example, while MDD’s broad impact on health is well established [3,4,5,6,7,8,9,10], few studies have investigated whether specific MDD subtypes are differentially associated with particular impairments. For subtyping frameworks to be clinically useful, they need to demonstrate consistent associations with clinically relevant outcomes, such as physical functioning, cognitive ability, and daily role performance. However, differences in how subtypes are defined and compared across studies may contribute to inconsistent findings, underscoring the need for a clearer synthesis of the evidence and evaluation of subtyping approaches to determine whether existing models meaningfully reflect differences in health status and treatment needs.

### 1.2. Purpose of Review

Therefore, this review synthesises the literature on some major MDD subtypes, the methods used to define them, and their associations with physical, cognitive, social, and functional health outcomes. By examining these associations, plus the consistency of the subtyping approaches used across studies, this study aims to clarify the current state of the evidence and explore the extent to which existing models of MDD diagnosis differentiate patients in clinically meaningful ways. In doing so, this review aims to identify key methodological challenges and inform future research to improve the clinical utility of MDD subtyping frameworks.

## 2. Materials and Methods

### 2.1. Search Strategy and Selection Criteria

This review was conducted as a systematic review in accordance with the PRISMA 2020 guidelines [26]. A retrospective protocol was registered with the Open Science Framework (OSF) and is available at: https://osf.io/pkr7b. The registration includes the review protocol and full search strategy (accessed on 30 April 2025).

A systematic search was conducted in PubMed to identify eligible studies. The search strategy was limited to peer-reviewed studies published in English between 2014 and 2025, so as to capture the most recent developments in MDD subtyping, and to ensure alignment with contemporary diagnostic frameworks (i.e., the current DSM and ICD), enhancing the comparability across studies and the relevance of findings to current clinical research. The English-language restriction aimed to minimise translation inconsistencies that could introduce bias. The search strategy was developed using keyword mapping from previous reviews and was refined iteratively to ensure comprehensive coverage. Boolean operators and truncation were used to maximise retrieval, incorporating free-text terms related to MDD subtypes and health outcomes across physical, cognitive, social, and functional health domains. These domains were selected based on their relevance to MDD-related impairment and their consistent inclusion in prior reviews. To enhance sensitivity, synonyms and alternative spellings were included. A full list of search terms is provided in Appendix A.

Studies were eligible if they included adults aged between 18 and 65 years, with a diagnosis of MDD confirmed through current DSM criteria using validated diagnostic interviews or clinician diagnosis. Studies had to classify participants into subtypes of MDD, using either DSM-defined specifiers (e.g., melancholic, atypical, anxious, mixed, undifferentiated), validated clinical tools (e.g., the CORE Index for Melancholia or the Hamilton Depression Rating Scale [HAMD] for anxious depression), or data-driven methods such as latent class analysis or cluster analysis. Studies were included if subtypes were clearly defined and examined in relation to health-related impairment, either through comparisons between subtypes or between subtypes and healthy controls. Health outcomes needed to be assessed using validated measurement tools (e.g., standardised scales or clinician ratings) or relevant biological markers (e.g., body mass index [BMI], glucose levels). Studies were excluded if they did not use these inclusion criteria, if they categorised MDD only by severity levels without distinct subtypes, or were retrospective studies, review articles, editorials, case reports, and studies without original data (Appendix B details the inclusion/exclusion criteria).

### 2.2. Screening and Data Extraction

The primary reviewer (JM) independently screened all records, while a second reviewer screened a randomly selected 20% of records to ensure adherence to the inclusion criteria. Discrepancies were resolved through discussion, and Supplementary Materials were consulted when necessary to clarify methodological details. This approach aligns with the recommendations for minimising selection bias and improving reproducibility in systematic reviews, and has been shown to enhance accuracy and reduce errors in study selection [27]. Titles and summaries were initially screened on PubMed for relevance before relevant records were imported into EndNote for reference management. Abstracts were then screened, and if eligibility remained unclear, the full text was reviewed for further evaluation. Full-text articles were then assessed against inclusion criteria to determine final eligibility. A structured data extraction form was used to systematically capture the key study characteristics for all included studies, including author, year, country, study design, sample size, population details, subtype classification methods, and assessed health outcomes.

Risk of bias was assessed for all included studies using the Newcastle–Ottawa Scale (NOS) [28], which evaluates selection, comparability, and outcome domains. One reviewer (JM) conducted the assessment, and scores were tabulated across subdomains to provide an overall appraisal of study quality. These assessments were used to describe the strength and limitations of included studies but were not used to exclude studies or weight findings in synthesis.

### 2.3. Data Synthesis

Studies were categorised into four health domains: physical, cognitive, social, and functional. These domains were derived by reviewing established health-related quality of life (HRQoL) measures, including the World Health Organization Quality of Life (WHOQOL) measure [29], the MOS 36-Item Short-Form Health Survey (SF-36) [30], and the World Health Organization Disability Assessment Schedule (WHODAS 2.0) [31], and identifying common core domains relevant to MDD. The final four domains were selected based on their consistent inclusion across measures and their established significance in the literature on MDD-related impairment [32,33,34,35]. Each domain was considered separately to reflect distinct aspects of impairment in MDD. Physical health was analysed independently due to its well-documented association with MDD, particularly in relation to factors such as metabolic health, cardiovascular risk, and lifestyle behaviours [36,37]. Included studies assessed outcomes such as BMI, waist circumference, glucose levels, lipid profiles, or other relevant biological or lifestyle indicators, using objective measures or validated self-report tools.

Cognitive functioning was distinguished as a separate domain given its central role in MDD-related impairment, particularly in memory, attention, and executive function [38,39,40]. Studies in the cognitive domain assessed outcomes using standardised neuropsychological tests that evaluated key areas of functioning, including memory, attention, executive function, and processing speed, using validated tools commonly employed in clinical and research settings.

Social and functional impairments were also categorised separately to better reflect distinct patterns of impairment in MDD. Social dysfunction was defined as difficulties with interpersonal functioning, social withdrawal, or reduced social support [41], and studies were included if they assessed outcomes such as social support, relationship quality, or social adaptation, typically via validated self-report measures. Functional impairment referred to limitations in work, independent living, and daily responsibilities [42], and included both self-reported and clinician-rated outcomes reflecting day-to-day functioning or disability.

Due to variability in study designs, subtype definitions (including both DSM-defined and data-driven methods), and outcome measures, the findings were primarily synthesised narratively. Where relevant, conceptual comparisons were made, but interpretations were framed within the context of how subtypes were defined and operationalised in each study.

Limited quantitative synthesis was possible for physical health outcomes, where a subset of studies reported effect estimates (e.g., odds ratios, regression coefficients) and used comparable outcome measures and reference groups. Where possible, these values were extracted directly from the studies or calculated using available summary statistics (e.g., means, standard deviations, or confidence intervals). In cases where similar metrics were used across studies, unweighted averages of standardised effect sizes were calculated to summarise trends. However, due to the small number of studies meeting these criteria and the substantial variability in how subtypes and outcomes were defined, no formal meta-analytic modelling was conducted. Heatmaps were used as a descriptive tool to visually summarise the direction and relative strength of effects but should not be interpreted as reflecting pooled or statistically integrated results.

Cognitive, social, and functional outcomes are presented descriptively in narrative form and are supported by tables using directional indicators to reflect general trends. The tables are intended to support interpretation rather than quantify effect sizes.

## 3. Results

**Figure 1:**
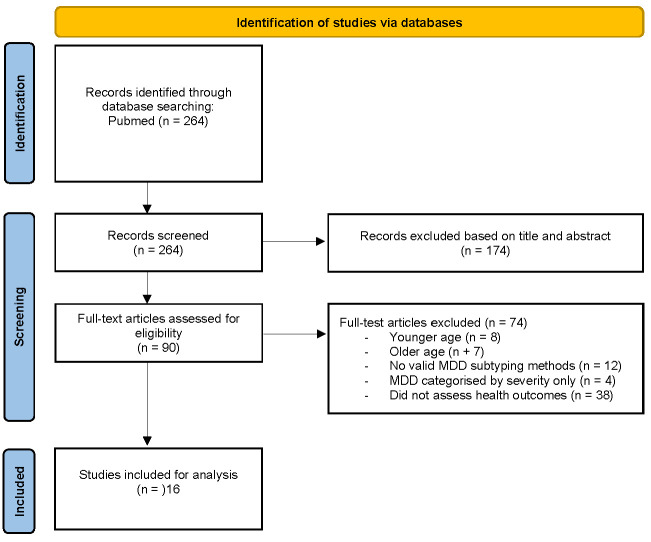
PRISMA flow diagram of study selection.

A search of PubMed returned 264 citations. Titles and abstracts were screened for relevance to MDD subtypes and associated health outcomes, reducing the selection to 90 full-text articles for further evaluation. After reviewing the full text of 90 articles, 74 were excluded for the following reasons: younger age, older age, lack of valid MDD subtyping, MDD subtyping by severity only, and lack of health outcome assessment. This resulted in a final selection of 16 studies for inclusion in this review. The screening process is illustrated in a PRISMA flow diagram (Figure 1).

### 3.1. Study Characteristics

The included studies comprised eight longitudinal (ranging from 8 weeks to 5.5 years) and eight cross-sectional studies, examining the short- and long-term impacts of MDD subtypes on health outcomes. Studies were conducted in China, The Netherlands, Switzerland, the USA, Spain, Germany, Taiwan, and Hong Kong, reflecting a concentration of research in Asia, North America, and Europe. Sample sizes ranged from 88 to 3054, drawn from both clinical and community populations. Participants ranged in age from 18 to 70 years, with mixed-gender samples. Detailed study characteristics are presented in Table 1.

### 3.2. Risk of Bias

The average NOS ratings across the included studies were 3.3/4 for selection, 1.9/2 for comparability, and 2.1/3 for outcome quality. Most studies were strong in selecting exposure and comparator groups, though many lacked a healthy control group. While most adjusted for key confounders, control for medication use and other potential sources of bias was inconsistent. Follow-up quality also varied, with eight studies relying on cross-sectional designs, limiting causal inference. Smaller studies potentially lacked sufficient statistical power to detect meaningful differences. Specifically, four studies had total sample sizes below 250, and an additional four studies had subgroups with fewer than 100 participants (Appendix C). Taken together, these methodological limitations, particularly the absence of healthy control groups and inconsistent adjustment for confounders, reduced comparability across studies and limited the extent to which generalisable conclusions could be drawn about subtype-related differences.

### 3.3. Subtyping Methods and Identified MDD Subtypes

Across the included studies, DSM-defined classifications were used in twelve studies, while data-driven methods were used in four studies. DSM-based approaches relied on structured clinical interviews, such as the Structured Clinical Interview for DSM Disorders (SCID) [59] and the Mini International Neuropsychiatric Interview (MINI) [60], or clinician assessment based on key symptom profiles consistent with DSM specifiers [15]. Several studies supplemented DSM criteria with validated clinical tools to refine subtype classification: two studies used the Inventory of Depressive Symptomatology (IDS-30) for atypical depression [61], four used the Hamilton Depression Rating Scale (HAMD-17) anxiety/somatization subscale (cutoff ≥ 7) for anxious depression [62], and three used the CORE Index for Melancholia (cutoff ≥ 7) for melancholic depression [63].

The most commonly examined DSM-based subtypes were melancholic (*n* = 7), atypical (*n* = 5), and anxious depression (*n* = 4), with four studies investigating more than one subtype. Additional classifications included mixed (*n* = 1) and undifferentiated subtypes (*n* = 3). Of the DSM-based studies, six included healthy control groups, allowing for a direct comparison of depression subtype individuals with non-depressed individuals.

In contrast, four studies used data-driven methods including LCA and cluster analysis to derive subtypes that reflect different symptom profiles from those identified using DSM-based classifications. All four studies included healthy control groups. These approaches identified novel subgroups of MDD, including cognitively preserved vs. impaired (K-means clustering); typical, atypical, moderate, severe melancholic, and severe atypical (LCA); and emotion-based clusters, including least impaired, generalised deficit, and an intermediate subtype.

### 3.4. Findings by Health Outcomes

#### 3.4.1. Physical Health

**Figure 2:**
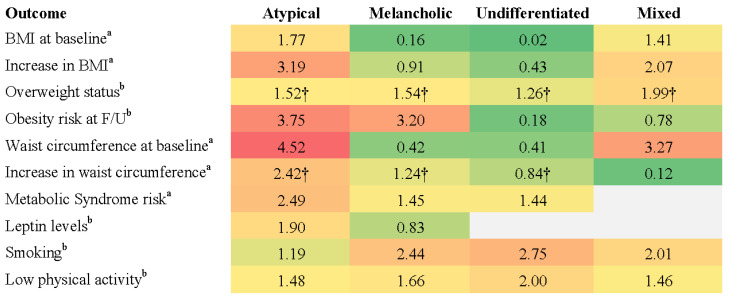
Effect sizes for physical health outcomes across DSM-defined MDD subtypes. *Note:* Heatmap displays effect sizes comparing DSM-defined MDD subtypes with non-MDD control groups across various physical health outcomes. Higher values (red) indicate stronger associations with adverse physical health indicators relative to reference group, while lower values (green) indicate weaker associations. Superscripts denote effect size type: ^a^ = beta coefficient; ^b^ = odds ratio. Cells marked with † represent pooled effect sizes, which were calculated by averaging unweighted values across studies due to variation in sample sizes and statistical methods. Values without † reflect single-study findings.

Physical health outcomes were assessed in five studies. Three studies used DSM-defined subtypes, while two employed data-driven classification methods. All five studies compared the outcomes to healthy controls. Despite some methodological variation, the three DSM-based studies shared sufficient consistency in subtyping methods, outcome measures, and comparator groups to support a limited quantitative synthesis. Unweighted pooled effect sizes were calculated where comparable metrics were reported and were visually summarised in a heatmap (Figure 2). No meta-analytic modelling was conducted. Studies using data-driven approaches were excluded from the heatmap due to differences in classification methods and the limited availability of comparable physical health outcomes.

Using DSM-defined subtypes, Lasserre et al. [46] found that individuals with atypical MDD had the highest baseline BMI (*M* = 26.8, *SD* = 4.1) and a significant increase over 5.5-year follow-up compared to healthy controls (*β* = 3.19, 95% CI [1.50, 4.88], *p* < 0.001), while no significant changes were observed for other DSM subtypes. Atypical MDD was also associated with the highest obesity risk among subtypes (OR = 3.75, 95% CI [1.24, 11.35], *p* < 0.05) [46]. Using a data-driven approach, Lamers et al. [45] reported that individuals with severe atypical MDD had a significantly higher mean BMI (*M* = 28.3, *SD* = 5.8) than those with severe melancholic (*M* = 25.2, *SD* = 5.0) or moderate depression (*M* = 25.7, *SD* = 5.1, *p* < 0.0001). Notably, Lamers et al. [45] reported a larger effect size for the association between severe atypical MDD and BMI (OR = 3.30) than that observed for the DSM-defined atypical subtype in Lasserre et al. [46] (OR = 1.77). The key physical health outcomes associated with DSM-defined subtypes are summarised in Figure 2.

Lasserre et al. [43] found that atypical depression was associated with greater central adiposity, indicated by a larger increase in waist circumference over a 5.5-year follow-up (*β* = 2.41, 95% CI [1.19, 3.63], *p* < 0.001). Melancholic depression also showed a significant but moderate increase (*β* = 1.43, 95% CI [0.36, 2.51], *p* < 0.01). Using data-driven methods, Lamers et al. [45] reported that the severe atypical subtype had the highest prevalence of metabolic syndrome (31.1%), compared to 20.2% in the severe melancholic subtype and 19.3% in the moderate subtype.

DSM-defined atypical MDD was associated with a significant increase in fasting glucose levels over the 5.5-year follow-up (*β* = 143, 95% CI [49, 237], *p* < 0.01) and a higher risk of developing metabolic syndrome (OR = 2.49, 95% CI [1.30, 4.77], *p* < 0.01) relative to healthy controls [43]. Using a data-driven approach to define atypical MDD, Milaneschi et al. [44] found that it was linked to elevated leptin levels (OR = 1.90, 95% CI [1.51, 2.93] *p* < 0.001), a hormone involved in energy regulation and appetite control. Increased leptin levels have been implicated in the metabolic disturbances observed in atypical depression, potentially contributing to greater adiposity and insulin resistance.

Distinct lifestyle patterns were observed across subtypes. DSM-defined melancholic depression was linked to higher rates of smoking (OR = 2.44, 95% CI [1.83, 3.25]), low physical activity (OR = 1.66, 95% CI [1.27, 2.18]), and overweight status (OR = 1.55, 95% CI [1.17, 2.06]) [47]. In contrast, DSM-defined atypical depression was associated with overweight status (OR = 2.54, 95% CI [1.19, 5.43]) but not with smoking or low physical activity [47]. DSM-defined mixed depression was linked to both smoking (OR = 2.01, 95% CI [1.21, 3.34]) and overweight status (OR = 2.07, 95% CI [1.20, 3.59]), while DSM-defined undifferentiated depression had the strongest association with smoking (OR = 2.75, 95% CI [1.90, 3.99]) and was also linked to low physical activity (OR = 2.00, 95% CI [1.41, 2.83]) [47].

#### 3.4.2. Cognitive Functioning

Cognitive outcomes were reported in seven studies. Six studies used DSM-defined subtypes, while one used a data-driven approach to classify individuals as cognitively preserved or impaired using K-means cluster analysis. Across these studies, a range of cognitive domains were assessed, including memory, processing speed, attention, executive function, and social cognition. However, substantial variation in the cognitive measures used and in the comparison groups limited comparability. Although these methodological differences constrained the strength of cross-study conclusions, general patterns of association were still able to be identified, offering preliminary insights into cognitive profiles across MDD subtypes and potential differences between classification approaches.

Overall, cognitive impairments in MDD subtypes, particularly in melancholic depression, appeared substantial and were often supported by statistically significant group differences across multiple domains. Compared to physical health outcomes, cognitive deficits were less consistently quantified with effect sizes but were frequently described with detailed test performance and *p*-values, supporting the clinical relevance of these impairments.

Memory and Working Memory. Roca et al. [54] reported that patients with DSM-defined melancholic depression exhibited greater deficits in verbal working memory compared to non-melancholic patients, with significantly lower WAIS-I Digit Span Forward (*p* = 0.027) and WAIS-II Digit Span Backward scores (*p* = 0.049). In contrast, Lin et al. [51] found no significant differences in verbal working memory (Digit Span Backward) or immediate visual reproduction (WMS-R) between subtypes, although all subtypes performed significantly worse than controls (*p* < 0.001).

Lu et al. [53] reported no significant differences in memory performance between patients with atypical versus those with non-atypical depression, and Liu et al. [52] reported no significant memory differences between anxious and non-anxious depression patients. However, Duan et al. [49] found better memory performance in patients with anxious depression, with significantly higher scores in verbal memory (HVLT-R, *p* = 0.003) and visual memory (BVMT-R, *p* = 0.005) compared to patients with non-anxious depression.

Processing Speed and Executive Function. Patients with melancholic depression demonstrated the most severe processing speed deficits, performing significantly worse than atypical and undifferentiated depression patients on WAIS-R Digit Symbol Coding (*p* < 0.001) and Trail Making Test-A (TMT-A, *p* < 0.001) [51]. No significant differences were found between patients with atypical versus non-atypical depression, or anxious and non-anxious depression in processing speed [52,53]. Patients with melancholic depression also exhibited reduced executive function compared to patients with non-melancholic depression, with longer completion times on the Trail-Making Test-B (TMT-B, *p* = 0.050), slower response times on the Stroop Colour-Word Test (SCWT-I, *p* = 0.031; SCWT-II, *p* = 0.005), and reduced psychomotor speeds on the Finger Tapping Test (*p* = 0.034) [54].

Cognitive Flexibility, Problem-Solving, and Attention. Melancholic and atypical depression patients exhibited greater cognitive inflexibility, completing fewer categories and making more errors on the Modified Wisconsin Card Sorting Test compared to undifferentiated depression patients (*p* < 0.001) [51]. Patients with melancholic depression also showed greater problem-solving deficits compared to non-melancholic patients, with longer Tower of London (TOL) problem-solving times (*p* = 0.018) and longer TOL execution times (*p* = 0.043) [54]. Similarly, performance on the Tower of Hanoi was significantly worse in melancholic and atypical depression patients compared to undifferentiated depression patients (*p* < 0.001) [51].

Melancholic depression patients exhibited slower attention-switching abilities (*d* = 0.168, *p* < 0.01) and longer reaction times (*d* = 0.211, *p* < 0.01) compared to non-melancholic depression patients [48]. In verbal interference tasks, melancholic patients performed significantly worse in naming tasks under time constraints (*d* = 0.168, *p* < 0.01) [48]. Set-shifting difficulties were further demonstrated in the Trail-Making Test-B (TMT-B) completion times, which were significantly longer in melancholic depression patients compared to atypical depression and undifferentiated depression patients (*p* < 0.001) [51].

Verbal Fluency and Social Cognition. Verbal fluency impairments were most pronounced in melancholic depression patients, particularly in semantic fluency tasks, with significantly lower Animal Naming Task scores than patients with atypical depression (*p* < 0.001) [51]. Melancholic depression was also associated with slower reaction times when identifying happy faces (*d* = 0.16, *p* < 0.05) and longer priming times for happy faces (*d* = 0.19, *p* < 0.01) compared to non-melancholic depression [48]. In contrast, atypical depression was characterised by lower attention/vigilance (*p* = 0.042) and greater social cognition impairments (*p* = 0.035) compared to non-atypical depression [53].

Cognitive Outcomes Over Time. Cognitive recovery varied by MDD subtype. Non-melancholic depression patients showed greater improvements in executive function and problem-solving over six months compared to those with melancholic depression [54]. In another study, patients with atypical depression demonstrated the most substantial recovery within six weeks, with most cognitive domains normalising except for persistent planning deficits [51], compared to melancholic and undifferentiated subtypes. In contrast, melancholic depression was associated with enduring impairments in processing speed (WAIS-R Digit Symbol Coding) and set-shifting (TMT-B, Wisconsin Card Sorting Test), even after symptom remission [51]. Undifferentiated depression also showed persistent but milder deficits [51].

Duan et al. [49] reported that anxious depression patients showed significant cognitive improvements over eight weeks, particularly in verbal fluency, processing speed, verbal and visual memory, and executive function (all *p* < 0.001). However, residual deficits in attention and executive function remained when compared to healthy controls. Table 2 presents an overview of the cognitive impairments and strengths associated with each DSM-defined MDD subtype.

Guo et al.’s research [50] was the only study in the cognitive domain to use a data-driven classification, applying K-means cluster analysis to group individuals with MDD into cognitively impaired and cognitively preserved subtypes based on test performance. Over a six-month follow-up period, 76.3% of those in the impaired group remained cognitively impaired, even if they achieved symptomatic remission. While not directly comparable to DSM-based subtypes, these findings raise important questions about whether cognitive deficits in MDD are stable, trait-like features that precede depressive episodes or emerge as a consequence of illness.

#### 3.4.3. Social and Functional Outcomes

Studies examining social functioning and functional disability in relation to MDD subtypes were limited, with only two studies assessing social outcomes and two examining functional disability, one of which contributed to both domains. Subtyping methods varied and included DSM-defined specifiers (e.g., melancholic, anxious) as well as data-driven clustering approaches based on emotion-related measures. This methodological heterogeneity, combined with the small number of studies, limited the potential for direct comparisons or quantitative synthesis. Nonetheless, the findings provide valuable insight into how different classification approaches have been applied to examine functioning in MDD and offer preliminary evidence of how specific subtypes may relate to social and occupational outcomes.

Social impairments. Zhou et al. [56] found that participants with DSM-defined anxious depression reported significantly greater interpersonal difficulties, including trouble engaging in conversations (*p* < 0.001), making friends (*p* < 0.001), and following social norms (*p* < 0.001). These participants also experienced significantly lower social support, with reduced objective support (*p* = 0.002), subjective support (*p* = 0.002), and support utilisation (*p* = 0.048). Family dysfunction was more pronounced in this group, with significant impairments in problem-solving (*p* = 0.001), communication (*p* < 0.001), family roles (*p* < 0.001), affective responsiveness (*p* = 0.002), and overall family functioning (*p* < 0.001).

Day et al. [58] found that patients with DSM-defined melancholic depression reported significantly poorer social relationships (*p* = 0.03) and greater social skill deficits (*p* < 0.001) than those with non-melancholic depression. These impairments persisted at the 8-week follow-up, with melancholic depression patients continuing to report poorer social skills (*p* = 0.03). Melancholic depression was also associated with greater emotional distress and maladaptive coping strategies, including a stronger negativity bias (*p* = 0.03), lower emotional resilience (*p* < 0.001), and a greater tendency to rely on suppression as an emotion regulation strategy (*p* < 0.001) [58].

Chan et al. [55] identified three MDD subtypes using two-stage cluster analysis based on emotion-related measures, including emotional experience, expression, and regulation strategies. The resulting clusters reflected distinct symptom profiles: Cluster 1 (least impaired), Cluster 2 (characterised by generalised emotional deficits), and Cluster 3 (intermediate). Social functioning was assessed using the Social Adaptation Self-evaluation Scale (SASS) [64], with Cluster 2 exhibiting significantly greater impairment than both Cluster 1 (*d* = 1.39, *p* < 0.001) and Cluster 3 (*d* = 0.75, *p* = 0.001), indicating that emotion-based subtypes were associated with distinct levels of social functioning. Table 3 summarises the general patterns of association between MDD subtypes and social functioning outcomes across the studies reviewed.

Functional Disability. Both melancholic and anxious depression were associated with significant impairments in occupational and daily functioning, as reported across two studies using DSM-defined subtypes. Day et al. [58] found that individuals with melancholic depression had lower social and occupational functioning scores compared to those with non-melancholic depression (*p* < 0.001). Similarly, Lin et al. [57] found that individuals with DSM-defined anxious depression had significantly greater impairments in daily role performance, including lower global functioning scores (*p* = 0.029) and higher work-related impairment scores (*p* = 0.011) than those with non-anxious depression.

Both melancholic and anxious depression were associated with significantly lower QoL scores across multiple domains. Day et al. [58] found that, compared to non-melancholic depression participants, melancholic depression patients had significantly lower WHOQOL scores for overall QoL (*p* < 0.001), physical health (*p* = 0.01), and psychological well-being (*p* < 0.001). Similarly, Lin et al. [57] reported that individuals with anxious depression exhibited significantly greater psychological impairment (*p* = 0.020), poorer physical functioning (*p* < 0.001), and increased bodily pain (*p* = 0.001). See Table 4 for an overview of functional disability outcomes by subtype.

## 4. Discussion

This review synthesised findings across physical, cognitive, social, and functional domains to assess whether major MDD subtypes are associated with distinct health profiles. While some patterns emerged, substantial inter-study methodological variation limited conclusions. The discussion below reflects on these findings and the implications of the subtyping approaches used.

### 4.1. Physical Health and Metabolic Outcomes

Across the included studies, atypical depression (both DSM-defined and data-driven) was most consistently associated with metabolic dysfunction, including higher BMI, increased waist circumference, and the greater prevalence of metabolic syndrome. While the small number of studies and variability in subtyping approaches limited generalisability, this pattern suggests some convergence between classification methods in identifying subgroups with elevated physical health risk. Notably, a study using a data-driven approach [45] reported larger effect sizes for the associations between a severe atypical subtype and metabolic indicators than those observed for the DSM-defined atypical subtype [46]. This may reflect differences in symptom severity, as data-driven models often capture more severe or homogenous profiles. These findings also provide preliminary support for the potential utility of symptom-based clustering in identifying subgroups with distinct biological risk profiles.

However, evidence of metabolic disturbance was not limited to the atypical depression subtype. Lamers et al. [45] also identified elevated metabolic syndrome prevalence in a severe melancholic depression group derived through data-driven clustering, though to a lesser extent than in severe atypical cases. Similarly, Lasserre et al. [43] found increases in waist circumference over time in both the melancholic and atypical groups, suggesting that metabolic risk may not be exclusive to one subtype. This challenges assumptions about clear subtype-bound biological patterns and points to a more nuanced relationship between depressive features and metabolic health.

Rahe et al. [47] found that atypical depression was primarily associated with weight gain and obesity risk, while melancholic depression was linked to lower physical activity and higher smoking rates. These behavioural correlates may underlie some of the observed differences in metabolic risk, suggesting that while biological mechanisms, such as dysregulated appetite, weight gain, and inflammatory processes, may drive metabolic change in atypical depression [44], behavioural risk factors may play a larger role in melancholic presentations.

Together, these findings highlight preliminary but consistent evidence linking atypical depression to greater metabolic dysfunction, with some alignment between DSM-based and data-driven classifications. Differences in observed effect sizes suggest that data-driven subtyping may offer added value in identifying subgroups at elevated physical health risk. At the same time, the presence of metabolic disturbance in other subtypes, particularly melancholic depression, indicates that such risks are not exclusive to atypical profiles. Observed differences in behavioural correlates across subtypes further underscore the complexity of linking depressive subtype classifications with physical health outcomes.

### 4.2. Cognitive Outcomes

Findings across studies revealed mixed but suggestive evidence of there being differential cognitive impairment by MDD subtype, particularly in domains such as working memory, executive function, and processing speed. Melancholic depression was most frequently associated with cognitive deficits, but results varied depending on the subtyping approach. For instance, Roca et al. [54] defined melancholic depression using DSM criteria supported by the CORE Index and HAMD-17 scores and found greater verbal working memory deficits in melancholic depression compared to non-melancholic depression. In contrast, Lin et al. [51] relied solely on DSM-based subtyping without supplementary tools and found no significant cognitive differences between melancholic, atypical, and undifferentiated subtypes, though all performed worse than healthy controls. These discrepancies may reflect differences in the specificity and severity captured by subtyping methods. The use of the CORE Index by Roca et al. [54] may have captured key melancholic features, such as psychomotor disturbance, a known correlate of cognitive impairment [65], contributing to the observed differences.

Some studies also reported cognitive impairments in DSM-defined atypical depression, particularly in attention, vigilance, and social cognition [51,53]. The presence of overlapping cognitive deficits across subtypes challenges the assumption that cognitive impairment is specific to melancholic depression, raising questions about whether DSM-based classifications adequately capture neurocognitive heterogeneity in MDD and whether alternative models incorporating cognitive function are warranted.

Findings from Guo et al. [50] highlighted the potential value of subtyping MDD based on cognitive functioning rather than symptom profiles alone. Their data-driven classification identified stable cognitive impairment in a substantial proportion of patients, even after mood symptoms remitted. While not directly comparable to DSM-based subtypes, this suggests that cognitive dysfunction may reflect a trait-like characteristic in a distinct subgroup of individuals with MDD. Such an approach may offer greater predictive validity for long-term outcomes, particularly in areas like occupational functioning and quality of life. Integrating cognitive performance into subtyping frameworks may therefore help identify individuals at risk for persistent functional impairment, informing more targeted interventions beyond symptom reduction.

### 4.3. Social and Functional Impairment

Findings from a small number of studies using DSM-defined subtypes suggested that anxious and melancholic depression may be associated with more severe social and functional impairment compared to other subtypes. Individuals with anxious depression reported greater social withdrawal, interpersonal difficulties, and lower social support than those with non-anxious MDD [56]. However, Zhou et al. [56] acknowledged that anxious depression may not represent a distinct depressive subtype but rather a variant of MDD, given its clinical similarities to generalised anxiety disorder.

Melancholic depression was also associated with reduced social engagement, social skill deficits, and greater reliance on maladaptive coping strategies, such as suppression and negativity bias [58]. These impairments persisted after treatment, suggesting potential resistance to social recovery. In occupational settings, melancholic depression was linked to greater workplace disability, including lower productivity and an increased likelihood of workplace impairment [58]. However, given its strong association with executive dysfunction and psychomotor slowing [51], cognitive deficits may play a central role in occupational impairment rather than melancholic features themselves.

Only one study used a data-driven approach to examine social functioning. Chan et al. [55] identified emotion regulation difficulties as a key predictor of social dysfunction in MDD, suggesting that factors beyond traditional symptom-based subtyping may offer greater explanatory value. This highlights the potential value of alternative subtyping frameworks that classify individuals based on underlying characteristics, such as emotion regulation, which may better identify individuals at risk of severe interpersonal difficulties.

### 4.4. Limitations and Future Directions

Several limitations should be considered when interpreting the findings of this review. Most notably, there was substantial variability in how MDD subtypes were defined and operationalised across studies. While some used DSM-based specifiers supplemented with validated tools, others relied solely on structured interviews or applied data-driven clustering methods. These methodological inconsistencies complicated comparisons and limited conclusions about which subtyping approaches most effectively differentiated health outcomes.

Outcome measures varied widely across studies, particularly in the cognitive, social, and functional domains, and the diversity in instruments and reporting methods limited comparability and synthesis. In addition to variability in measurement tools, reporting practices differed, limiting direct comparisons of effect size magnitude or clinical significance across domains. Physical health outcomes were more often reported using standardised effect sizes and biological indicators, allowing for limited pooling and visual comparison. In contrast, cognitive outcomes were typically reported using group comparisons on individual neuropsychological tests with associated *p*-values, while social and functional outcomes were mostly reported using aggregate scores from self-report instruments, often without standardised effect sizes. This variability limited the ability to evaluate the relative strength or clinical significance of associations across domains, as differences in the reporting formats and statistical detail constrained cross-domain interpretation.

Additionally, comparison groups differed, with some studies using healthy controls, and others comparing subtypes within MDD samples, making it difficult to assess the magnitude and specificity of impairments. The small number of studies contributing to each domain, particularly social and functional outcomes, further limited the strength of conclusions. Additionally, sample sizes varied considerably (ranging from 88 to 3054), which may have influenced the robustness and generalisability of findings. Most studies also employed cross-sectional designs, restricting the ability to draw causal inferences or assess the stability of impairments over time.

Future research should aim to improve methodological consistency by adopting clearly defined subtyping criteria and using standardised outcome measures across health domains. Future research would also benefit from the more consistent reporting of effect sizes to support clearer cross-domain comparisons. In addition, studies should, where possible, include both healthy control and within-MDD comparison groups to clarify the specificity of subtype-related impairments. Furthermore, longitudinal research is needed to assess the stability of these impairments over time and their implications for clinical prognosis. The effects of confounding variables, such as diet, physical activity, and medication use, which may influence body weight, could also be included in future studies. Finally, future subtyping frameworks may benefit from combining biological, cognitive, and functional markers with symptom-based classifications to better identify individuals at risk of long-term disability and to improve the predictive validity of depression classification systems.

### 4.5. Clinical Implications

Although not aimed at defining the specific implications for everyday clinical practice, this review points out how some specific MDD subtypes are often accompanied by dysregulations, disorders, or illnesses. Clinicians need to be cognizant of the likelihood that these may occur, as outlined in the Discussion section.

## 5. Conclusions

This review examined the association between major depressive disorder (MDD) subtypes and health outcomes across physical, cognitive, social, and functional domains, while also critically evaluating the subtyping methods used. The findings indicated that some subtypes, particularly atypical, melancholic, and anxious depression, were differentially associated with specific patterns of impairment. However, these associations were not consistent across studies and often depended on how subtypes were defined and compared.

While DSM-based classifications remain widely used, findings from data-driven studies suggest that alternative subtyping frameworks may better capture meaningful variations in health outcomes. Some emerging models, including those incorporating cognitive or behavioural characteristics, show potential for improving the identification of individuals at risk of persistent functional impairment. Future classification systems should aim to integrate multiple health indicators, such as functional capacity, cognitive performance, behavioural characteristics, and biological markers, alongside symptom profiles, to enhance the clinical utility of MDD subtypes. Greater consistency in subtyping methods and outcome measurement will also be critical to advancing the field and informing the development of more targeted, evidence-based interventions.

## Figures and Tables

**Table 1:** Study characteristics.

Physical Health and Metabolic Outcomes							
Author (Year)	Country	Study Design	Sample Size	Population Characteristics	MDD Subtypes and Classification Method	Health Outcomes Assessed	Measures Used
Lasserre et al. (2017) [43]	Switzerland	Longitudinal cohort study (5.5-year follow-up)	2813 participants (179 atypical depression, 369 melancholic depression, 685 unspecified depression, 1580 non-MDD)	Adults (35–66 years old) from the CoLaus/PsyCoLaus population-based cohort	Melancholic, atypical, unspecified; DSM-IV criteria (DIGS) and symptom specifiers	Metabolic health, physical health	Fasting glucose, HDL cholesterol, triglycerides, systolic blood pressure, metabolic syndrome, leptin levels (ELISA assay), BMI, waist circumference
Milaneschi et al. (2017) [44]	The Netherlands	Cross-sectional observational	2270 participants (271 current severe typical, 521 current moderate, 270 current severe atypical, 711 remitted MDD, 497 HC)	Adults (18–65 years old) from The Netherlands Study of Depression and Anxiety (NESDA)	Atypical, typical, and moderate; LCA based on symptom profiles	Physical health	Leptin levels (ELISA assay), BMI, waist circumference
Lamers et al. (2016) [45]	The Netherlands	Longitudinal observational (6-year follow-up)	1248 participants (308 severe melancholic, 167 severe atypical, 173 moderate, 600 HC)	Adults (18–65 years old) with MDD (CIDI) recruited from the NESDA cohort	Severe melancholic, severe atypical, and moderate; LCA based on CIDI and IDS-SR symptom profiles	Somatic health, overall functioning	BMI, metabolic syndrome prevalence, waist circumference, fasting glucose, triglycerides, HDL cholesterol, blood pressure, WHODAS
Lasserre et al. (2014) [46]	Switzerland	Longitudinal cohort (5.5-year follow-up)	3054 participants (48 current atypical, 55 current melancholic, 31 current combined, 97 current unspecified, 1121 remitted MDD, 1702 non-MDD)	Adults (35–66 years old) from the CoLaus/PsyCoLaus population-based cohort	Melancholic and atypical; DSM-IV criteria (DIGS) and symptom specifiers	Physical health	BMI, waist circumference, fat mass (bioimpedance)
Rahe et al. (2016) [47]	Germany	Cross-sectional observational	1420 participants (503 melancholic depression, 43 atypical depression, 81 mixed depression, 196 undifferentiated depression, 597 HC)	Adults (35–65 years old) from the BiDirect Study	Melancholic, atypical, mixed, and undifferentiated; DSM-IV criteria (MINI) and IDS items for atypical features	Physical health	BMI, combined lifestyle index (0–4 unhealthy lifestyle factors)
**Cognitive Outcomes**							
**Author (Year)**	**Country**	**Study Design**	**Sample Size**	**Population** **Characteristics**	**MDD Subtyping Method**	**Health Outcomes Assessed**	**Measures Used**
Day et al. (2015a) [48]	iSPOT (USA, Australia, The Netherlands, New Zealand, South Africa)	Cross-sectional observational	1344 participants (339 melancholic depression, 669 non-melancholic depression, 336 HC)	Adults (18–65 years old) diagnosed with MDD (DSM-IV, MINI)	Melancholic; DSM-IV criteria and psychomotor disturbance (CORE score ≥ 7)	Cognitive function, emotional function	Standardised cognitive battery
Duan et al. (2021) [49]	China	Longitudinal observational (8-week follow-up)	1048 participants (328 anxious depression, 221 non-anxious depression, 499 HC)	Adults (18–55 years old) with MDD (MINI)	Anxious; HAMD-17 anxiety/somatization factor score ≥ 7	Cognitive function	HVLT-R, BVMT-R, SCWT, CPT
Guo et al. (2023) [50]	China	Longitudinal observational (6-month follow-up)	295 participants (91 MDD preserved cognition, 62 MDD impaired cognition, 142 HC)	Medication-free adults (18–55 years old) with MDD (SCID)	Cognitive subtypes (preserved vs. impaired cognition); K-means cluster analysis	Cognitive function	DSB, DSF, SCWT, TMT-A, TMT-B, SVF, VMT, WMS
Lin et al. (2014a) [51]	China	Prospective longitudinal	509 participants (142 melancholic depression, 76 atypical depression, 91 undifferentiated depression, 200 HC)	Adults (18–60 years old) with MDD (DSM-IV-TR)	Melancholic, atypical, and undifferentiated; DSM-IV structured clinical interviews	Cognitive function	TMT-A, DSC (WAIS-RC), DSF (WAIS-RC), DSB (WAIS-RC), WCST-M, TMT-B, TOH, AN, IVR (WMS-RC)
Liu et al. (2019) [52]	China	Cross-sectional observational	214 participants (138 anxious depression, 76 non-anxious depression); no HC	Adults (18–65 years old) with MDD (DSM-5)	Anxious; HAMD-17 anxiety/somatization factor score ≥ 7	Cognitive function, social and occupational function	MCCB, GAF
Lu et al. (2023) [53]	China	Cross-sectional observational	353 participants (101 atypical depression, 252 non-atypical depression); no HC	Adults (16–60 years old) with MDD (MINI)	Atypical; DSM-5 criteria and Inventory of Depressive Symptomatology (IDS-30)	Cognitive function, QoL	MCCB, QOL-6
Roca et al. (2015) [54]	Spain	Longitudinal observational (6-month follow-up)	88 participants (25 melancholic depression, 63 non-melancholic depression); no HC	Adults (18–55 years old) with MDD (DSM-IV-TR)	Melancholic; DSM-IV-TR, CORE Index for Melancholia, and HAMD-17 score ≥ 17	Cognitive function	TMT-A, TMT-B, DSF (WAIS-III), DSB (WAIS-III), SCWT, TOL DX, FAS, SVF (Animals), FTT
**Social and Functional Impairment**							
**Author (Year)**	**Country**	**Study Design**	**Sample Size**	**Population** **Characteristics**	**MDD Subtyping Method**	**Health Outcomes Assessed**	**Measures Used**
Chan et al. (2023) [55]	Hong Kong	Cross-sectional observational	200 participants (150 MDD patients, 50 controls)	Adults (18–65 years old) with MDD (SCID)	Social subtypes; two-stage cluster analysis based on emotion-related measures (TEPS, TAS, ERQ)	Social functioning	Social Adaptation Self-Evaluation Scale (SASS)
Zhou et al. (2023) [56]	China	Cross-sectional observational	809 participants (326 anxious depression, 483 non-anxious depression); no HC	Adults (19–23 years old) with MDD (MINI)	Anxious; HAMD-17 anxiety/somatization factor score ≥ 7	Family functioning, social support, interpersonal problems	Family Assessment Device (FAD), Social Support Rating Scale (SSRS), Interpersonal Relationship Integrated Diagnostic Scale (IRIDS)
Lin et al. (2014b) [57]	Taiwan	Cross-sectional observational	174 participants (141 anxious depression, 33 non-anxious depression); no HC	Adult inpatients (18–70 years old) with MDD (SCID)	Anxious and non-anxious depression; (HAMD-17) anxiety/somatization factor score ≥ 7	Pain, QoL, daily functioning	SF-36 Body Pain Index (BPI), SF-36 Physical Component Summary (PCS), SF-36 Mental Component Summary (MCS), Global Assessment of Functioning (GAF), Work and Social Adjustment Scale (WSAS)
Day et al. (2015b) [58]	iSPOT (USA, Australia, The Netherlands, New Zealand, South Africa)	Longitudinal observational study (8-week follow-up)	1008 MDD participants (339 melancholic, 667 non-melancholic); no HC	Adults (18–65 years old) with MDD (DSM-IV, MINI)	Melancholic; DSM-IV criteria and psychomotor disturbance (CORE score ≥ 7)	Functional capacity, distress and coping, personality, emotion regulation	Social and Occupational Functioning Assessment Scale (SOFAS), World Health Organization Quality of Life (WHOQOL), Brief Risk-Resilience Index for Screening (BRISC), Satisfaction with Life Scale (SWLS), NEO-Five Factor Inventory (NEO-FFI), Emotion Regulation Questionnaire (ERQ)

*Abbreviations:* AN—Animal Naming; BVMT-R—Brief Visuospatial Memory Test—Revised; CPT—Continuous Performance Test; DSB—Digit Span Backward; DSF—Digit Span Forward; DSC—Digit Symbol Coding; FAS—Controlled Oral Word Association Test; FTT—Finger Tapping Test; GAF—Global Assessment of Functioning; HC—Healthy Controls; HVLT-R—Hopkins Verbal Learning Test—Revised; iSPOT—International Study to Predict Optimised Treatment for Depression; IVR—Immediate Visual Reproduction (WMS-RC); MCCB—MATRICS Consensus Cognitive Battery; QOL-6—6-item Quality of Life Scale; SCWT—Stroop Color Word Test; SVF—Semantic Verbal Fluency; TMT-A—Trail-Making Test Part A; TMT-B—Trail-Making Test Part B; TOH—Tower of Hanoi; TOL DX—Tower of London—Drexel Version; WAIS-RC—Wechsler Adult Intelligence Scale—Revised, Chinese Version; WMS—Wechsler Memory Scale; WCST-M—Wisconsin Card Sorting Test—Modified.

**Table 2:** Summary of cognitive functioning across DSM-defined MDD subtypes.

DSM-Defined MDD Subtype	Group Comparison (*n*)	Outcome	*p*-Value
Melancholic [54]	Mel. (25) vs. NM (63)	↓ verbal working memory	
		DSF	0.027
		DSB	0.049
		↓ executive function	
		TMT-B	0.05
		SCWT-I	0.031
		SCWT-II	0.005
		↓ psychomotor speed	
		FTT	0.034
		↓ problem-solving	
		TOL DX problem-solving	0.018
		TOL DX execution	0.043
[48]	Mel. (339) vs. NM (669)	↓ attention-switching	<0.01
[51]	Mel. (142) vs. Atypical (76); Undiff. (91)	↓ processing speed	
		DSC	<0.001
		TMT-A	<0.001
		↑ cognitive inflexibility	
		WCST-M	<0.001
		↓ semantic fluency	
		AN	<0.001
Atypical [51]	Atypical (76) vs. Mel. (142); Undiff. (91)	↑ cognitive inflexibility	
		WCST-M	0.001
[53]	Atypical (101) vs. Non-atypical (252)	↓ attention/vigilance	0.042
		↑ social cognition impairments	0.035
Anxious [49]	Anxious (328) vs. Non-anxious (221)	↑ verbal memory	
		HVLT-R	0.003
		↑ visual memory	
		BVMT-R	0.005

*Note:* This table summarises the findings where one DSM-defined MDD subtype demonstrated greater cognitive impairment than other subtypes. Studies reporting equivalent impairment across subtypes are not included. ↓ denotes lower performance or a reduction in a given domain, while ↑ denotes higher performance or an increase in a given domain. Increases may represent either improvements (e.g., better memory) or impairments (e.g., greater cognitive inflexibility), depending on the specific outcome measured. *Abbreviations:* AN—Animal Naming; BVMT-R—Brief Visuospatial Memory Test—Revised; DSB—Digit Span Backward; DSF—Digit Span Forward; DSC—Digit Symbol Coding; FTT—Finger Tapping Test; HVLT-R—Hopkins Verbal Learning Test—Revised; Mel.—Melancholic; NM—Non-melancholic; SCWT—Stroop Color Word Test; TMT-A—Trail-Making Test Part A; TMT-B—Trail-Making Test Part B; TOL DX—Tower of London—Drexel Version; Undiff.—Undifferentiated; WCST-M—Wisconsin Card Sorting Test—Modified.

**Table 3:** Summary of social functioning across MDD subtypes.

MDD Subtype	Group Comparison (*n*)	Outcome	*p*-Value
Generalised Emotional Deficits (Data-driven) [55]	Cluster 2 (66) vs. Cluster 1 (50); Cluster 3 (34)	↓ social adaptation SASS	<0.001
Anxious [56]	Anxious (326) vs. Non-anxious (483)	↑ interpersonal difficulties engaging in conversations making friends following social norms ↓ social support objective support subjective support support utilisation ↑ family dysfunction problem-solving communication family roles affective responsiveness overall family functioning	<0.001 <0.001 <0.001 0.002 0.002 0.048 <0.001 <0.001 <0.001 0.002 <0.001
Melancholic [58]	Mel. (339) vs. NM (667)	↓ social relationships ↑ social skill deficits ↑ emotional distress and maladaptive coping strategies negativity bias emotional resilience suppression as an emotion regulation strategy	0.03 <0.001 0.03 <0.001 <0.001

*Note:* This table summarises the findings where one MDD subtype demonstrated greater social impairment than other subtypes. Studies reporting equivalent impairment across subtypes are not included. Only one study used a data-driven approach in this domain. ↓ denotes reduced functioning or impairment; ↑ denotes an increase in a given trait, which may indicate either improvement or worsening depending on the outcome. For example, “↑ social skills deficits” indicates greater impairment, while “↑ social support” (if present) would indicate better functioning. *Abbreviations:* Mel.—Melancholic; NM—Non-melancholic; SASS—Social Adaptation Self-evaluation Scale.

**Table 4:** Summary of functional impairments across DSM-defined MDD subtypes.

DSM-Defined MDD Subtype	Group Comparison (*n*)	Outcome	*p-*Value
**Anxious** [57]	Anxious (141) vs. Non-anxious (33)	↓ global functioning ↑ work-related impairment ↑ psychological impairment ↓ physical functioning ↑ bodily pain	0.029 0.011 0.020 <0.001 0.001
**Melancholic** [58]	Mel. (339) vs. NM (667)	↑ impairments in social and occupational functioning ↓ overall QoL ↓ physical health ↓ psychological well-being	<0.001 <0.001 0.01 <0.001

*Note:* This table summarises the findings where one DSM-defined MDD subtype demonstrated greater functional impairment than other subtypes. Studies reporting equivalent impairment across subtypes are not included. ↓ denotes reduced functioning or impairment; ↑ denotes an increase in a given trait, which may indicate either improvement or worsening depending on the outcome. For example, “↑ work-related impairment” indicates worsening, while “↑ psychological well-being” (if present) would indicate improvement. *Abbreviations:* Mel.—Melancholic; NM—Non-melancholic; QoL—Quality of life.

## Data Availability

Data are available in this manuscript and the reviewed papers.

## Appendix A. Search Strategy

The search strategy was developed to identify studies evaluating MDD subtypes and their associated health outcomes in the PubMed database from 2014 to 2025 (inclusive). Boolean operators and truncation were used to maximise retrieval, incorporating free-text terms and controlled vocabulary where applicable.

**Search Term**	**Field**	**Yield**
1. Depression OR MDD OR “major depressive disorder *” OR “major depressive episode” OR MDE	Title	80,105
2. subtype * OR Depression subtype * OR Subtypes major depressive disorder * OR Subtype major depressive disorder * OR “Subtypes of depression” OR “clinical phenotype *” OR “symptom cluster *” OR “symptom profile *” OR melancholic OR Melancholic depression OR atypical OR “atypical depression” OR “anxious depression” OR “Latent class analysis” OR “Cluster analysis” OR “factor analysis” OR “Data-driven” OR “latent profile analysis” OR “latent transition analysis”	Title	55,999
3. “Health outcome *” OR outcome * OR “Clinical outcome *” OR “medical outcomes” OR “risk factor *” OR consequence * OR “health consequences” OR predict * OR “quality of life” OR “function *” OR impair * OR “Functional impairment”	Title/Abstract	5,540,335
4. Physical OR somatic OR cardiovascular OR cardiometabolic OR cardio-metabolic OR Diabetes OR Pain OR Fatigue OR Sleep * OR insomnia OR “Blood pressure” OR hyperglycemia OR hyperglycaemia OR inflammation OR “inflammatory marker” OR “metabolic syndrome” OR obesity OR BMI OR “body mass index” OR “lifestyle factor *” OR “health behaviour *” OR “health behavior” OR lifestyle OR cognitive OR “cognitive impairment” OR “cognitive function *” OR “social impairment” OR “social function *” OR “occupational function *”	Title/Abstract	2,744,490
5. 1 and 2 and 3 and 4		264

* Filters: from 2014–2025.

## Appendix B. Inclusion and Exclusion Criteria

The following table outlines the inclusion and exclusion criteria applied during study selection. Studies were eligible if they examined subtypes of major depressive disorder (MDD) and their associated health outcomes, using validated diagnostic and outcome assessment methods. Exclusion criteria were applied to ensure relevance to the research question and to maintain methodological rigour.

**Category**	**Inclusion Criteria**	**Exclusion Criteria**
Population	- Adults (18–65 years) diagnosed with MDD using DSM or ICD criteria, confirmed through validated diagnostic interviews (e.g., SCID, MINI) or clinician diagnosis.	- Studies on adolescents (<18 years) or late-life depression (>65 years).
Subtyping Method	- Subtypes classified using data-driven methods (e.g., LCA, clustering), DSM symptom criteria/specifiers, or validated clinical assessment tools (e.g., HAMD, CORE Index for Melancholia).	- Studies that did not classify MDD subtypes using data-driven methods, DSM symptom criteria/specifiers, or validated clinical assessment tools. - Studies that categorised MDD only by severity levels (mild/moderate/severe) without distinct subtypes
Health Outcomes	- At least one health-related outcome assessed, categorised into physical health, mental health, cognitive function, social/occupational functioning, functional disability, or quality of life.	- Studies that did not assess health outcomes.
Outcome Measures	- Use of validated tools for assessing health outcomes (e.g., SF-36, WHOQOL, WHODAS, HAMD, neurocognitive tests) or relevant biological markers (e.g., BMI, metabolic syndrome criteria).	- Studies that did not use validated health outcome measures.
Study Design	- Longitudinal studies - Cross-sectional studies	- Review articles, editorials, case reports, or studies without original data.
Language	- English	

*Abbreviations:* MDD—Major Depressive Disorder; DSM—Diagnostic and Statistical Manual of Mental Disorders; ICD—International Classification of Diseases; SCID—Structured Clinical Interview for DSM Disorders; MINI—Mini-International Neuropsychiatric Interview; LCA—Latent Class Analysis; HAMD—Hamilton Depression Rating Scale; SF-36—Short Form Health Survey; WHOQOL—World Health Organization Quality of Life; WHODAS—World Health Organization Disability Assessment Schedule; BMI—Body Mass Index.

## Appendix C. Study Quality Assessment (Newcastle–Ottawa Scale)

To evaluate the methodological quality of included studies, the Newcastle–Ottawa Scale (NOS) was used to assess the selection, comparability, and outcome domains. Each study was rated based on its ability to minimise bias in participant selection, control for confounders, and ensure robust outcome measurement. Higher scores indicate stronger methodological quality.

	**Number of Stars**			
**Study (Year)**	**Selection ***	**Comparability ^†^**	**Exposure ^‡^**	**Overall**
Chan et al. (2023) [55]	3	2	1	6
Day et al. (2015a) [48]	4	2	3	9
Day et al. (2015b) [58]	3	2	1	6
Duan et al. (2021) [49]	4	2	3	9
Guo et al. (2023) [50]	4	2	3	9
Lamers et al. (2016) [45]	4	2	3	9
Lasserre et al. (2014) [46]	4	2	3	9
Lasserre et al. (2017) [43]	4	2	3	9
Lin et al. (2014) [51]	2	2	1	5
Lin et al. (2014b) [57]	4	2	3	9
Liu et al. (2019) [52]	2	2	1	5
Lu et al. (2023) [53]	2	2	1	5
Milaneschi et al. (2017) [44]	4	2	1	7
Rahe et al. (2016) [47]	4	2	1	7
Roca et al. (2015) [54]	3	2	3	8
Zhou et al. (2023) [56]	2	1	3	6
**Average ratings**	**3.3**	**1.9**	**2.1**	**7.4**

* Maximum 4 stars. ^†^ Maximum 2 stars. ^‡^ Maximum 3 stars.
